# miR-96-5p: A potential diagnostic marker for gestational diabetes mellitus

**DOI:** 10.1097/MD.0000000000025808

**Published:** 2021-05-28

**Authors:** Xinyang Yu, Zhengfei Liu, Jie Fang, Hongbo Qi

**Affiliations:** Department of Obstetrics and Gynecology, The First Affiliated Hospital of Chongqing Medicine University, Chongqing, China.

**Keywords:** biomarker, gestational diabetes mellitus, microRNA, trophoblast

## Abstract

MicroRNAs play important roles in gestational diabetes mellitus (GDM), and this study aimed to elucidate the clinical significance of miR-96-5p in diagnosing GDM.

There are 123 pregnant women diagnosed with GDM and 123 healthy pregnant women were enrolled as control participants. Placenta and plasma samples from the patients and control participants were collected, and quantitative reverse transcription polymerase chain reaction (RT-qPCR) was performed to determine miR-96-5p expression levels. Moreover, a receiver operating characteristic (ROC) curve was established to evaluate the significance of miR-96-5p in diagnosing GDM. HRT-8/SVneo trophoblasts were cultured under high glucose conditions and treated with miR-96-5p mimics, and cell viability was examined.

miR-96-5p levels were significantly decreased in both the placenta and plasma samples of patients with GDM. The ROC curve indicated that miR-96-5p can serve as a diagnostic biomarker for GDM with high sensitivity and specificity. Moreover, miR-96-5p levels were markedly low under high glucose conditions, and the overexpression of miR-96-5p increased the viability, respectively, of trophoblasts in vitro.

miR-96-5p may participate in the pathogenesis of GDM by exerting effects on the viability of trophoblasts.

## 1 Introduction

Gestational diabetes mellitus (GDM) is a common obstetric disease with a high incidence rate among pregnant women.^[1–3]^ GDM affects approximately 7% of all pregnancies varying between 1% and 14% depending on the population studied and the diagnostic criteria used.^[4]^ The total incidence of GDM in mainland China is approximately 15%,^[5]^ but it can also be as high as 20% in some areas such as Peking University First Hospital and Tianjin.^[6,7]^ A clear pathogenesis of GDM has not been fully elucidated, and in some cases, GDM can progress into type 2 diabetes mellitus.^[2,8]^ Therefore, further studies on the underlying mechanism of GDM would be beneficial in identifying new biomarkers and therapeutic targets.

MicroRNAs are small non-coding RNAs of approximately 20 to 22 nucleotides (nt). They are reportedly ubiquitous in multicellular organisms.^[9–11]^ Mature microRNAs can post-transcriptionally regulate gene expression by mediating the silencing of their target mRNA, which consequently affect the synthesis of target proteins.^[12]^ In recent years, microRNAs have been associated with the development of many human diseases, including cancers,^[13,14]^ autoimmune diseases,^[15,16]^ and cardiovascular disease.^[17]^

The regulatory roles of microRNAs in obstetric diseases, including GDM, have been previously reported.^[18–21]^ In a study on the roles of miRNAs in GDM, miRNA expression profiling was performed, and miR-96-5p was reported as one of the most significantly downregulated miRNAs in the placentas of patients with GDM.^[22]^ To confirm the potential function and elucidate the regulatory mechanism of miR-96-5p in GDM, we collected placenta and plasma samples from patients with GDM, and measured the expression levels of miR-96-5p by using quantitative reverse transcription polymerase chain reaction (RT-qPCR). Moreover, the effects of miR-96-5p on the cell viability of trophoblast cells under high glucose conditions were also investigated. Our findings would provide a basis for miR-96-5p as a diagnostic biomarker and a therapeutic target for GDM.

## 2 Material and methods

### 2.1 Patients and samples

We enrolled a total of 246 participants, 123 of which were patients with GDM at the Department of Obstetrics and Gynecology, The First Affiliated Hospital, Chongqing Medical University, between May 2016 and September 2019. Women who were 24 to 28 weeks pregnant were examined for GDM. The diagnosis of GDM was conducted according to the diagnostic instructions for GDM established by the American Diabetes Association^[23]^: fasting plasma glucose ≥92 mg/dL (5.1 mmol/L) and/or 1-hour plasma glucose ≥180 mg/dL (10.0 mmol/L) and/or 2-hour plasma glucose ≥153 mg/dL (8.5 mmol/L) during the 75 g of oral glucose tolerance test (OGTT). The other 123 participants were healthy pregnant women, were enrolled as the control group, and were tested for tolerance to 75 g of OGTT; their plasma glucose values were normal. Patients with pregestational diabetes mellitus, abnormal liver and kidney function, malignant tumors, and systemic infections were excluded. Placenta samples were immediately stored in liquid nitrogen within 30 minutes after childbirth to reduce the risk of RNA degradation. Plasma samples from patients with GDM and control participants were also collected. Written informed consent from all participants were obtained. This study received support from the ethical committee of Chongqing Medical University.

### 2.2 Cell culture

HRT-8/SVneo trophoblast cells were passaged upon reaching 80% to 90% confluence; the cells from generations 3 to 5 were used for succeeding procedures. The control group was cultured in Dulbecco Modified Eagle Medium (DMEM) (Gibco, Waltham, MA) supplemented with 10% fetal bovine serum (FBS) (Gibco) and 1000 mg/mL glucose. The trophoblast cells cultured in DMEM and high glucose medium (Gibco) containing 4500 mg glucose/mL were considered the cells under high glucose conditions.

### 2.3 Cell transfection

HRT-8/SVneo cells were divided into 3 groups: control, high glucose + miR-96-5p mimics negative control (NC), and high glucose + miR-96-5p mimics. The miR-96-5p mimics and miR-96-5p NC were purchased from GenePharma (Shanghai, China). Transfection was performed using Lipofectamine 3000 (Invitrogen) according to the manufacturer's instructions.

### 2.4 RT-qPCR

The total RNAs in the placenta and plasma samples and trophoblast cells were extracted by using TRIzol (Invitrogen), and cDNA was reverse-transcribed and amplified by using PrimeScript OneStep RT-qPCR kit (Invitrogen) according to the manufacturer's instructions. RT-qPCR was performed as previously described (21): 10 minutes at 95 °C; 50 cycles of 10 seconds at 95 °C, 10 seconds at 55 °C, 5 seconds at 72 °C, 1 second at 99 °C, 15 seconds at 59 °C, and 1 second at 95 °C. U6, a housekeeping gene, was used as an internal control. miR-96-5p levels in each sample were normalized against U6 levels using the 2^−ΔΔCt^ method. Primers were purchased from Genscript (Nanjing, China), and the sequences of the primers used in this study are as follows: miR-96-5p forward and reverse: 5′-CCTTTGGCACTAGCACATTTTTG-3′and 5′-ACGCAAATTCGTGAAGCGTT-3′; U6 forward and reverse: 5′-CTCGCTTCGGCAGCACA-3′ and 5′-AACGCT TCACGAATTTGCGT-3′.

### 2.5 Cell proliferation assay

MTT assay was performed for cell proliferation analysis. Briefly, HRT-8/SVneo cells from different treatment groups were seeded into 96-well plates, added 10 μL MTT solution and incubated for 4 hours. Afterwards, the supernatant was discarded, and 100 μL dimethyl sulfoxide solution was added to each well. Optical density (OD) value at 490 nm wavelength was measured.

### 2.6 Statistical analysis

Statistical analysis was performed using GraphPad Prism 7.0 (San Diego, CA). All statistical data are presented as mean ± standard deviation. Data comparison was performed using Student *t* test (2 groups) or one-way analysis of variance (ANOVA) (3 groups). Correlation study was performed using Pearson coefficient analysis, and receiver operating characteristic (ROC) curves were established to determine the diagnostic significance of miR-96-5p. All experiments were performed in triplicate, and *P* < .05 was considered statistically significant.

## 3 Results

### 3.1 Decreased miR-96-5p expression levels in placenta and plasma samples from patients with GDM

**Figure 1:**
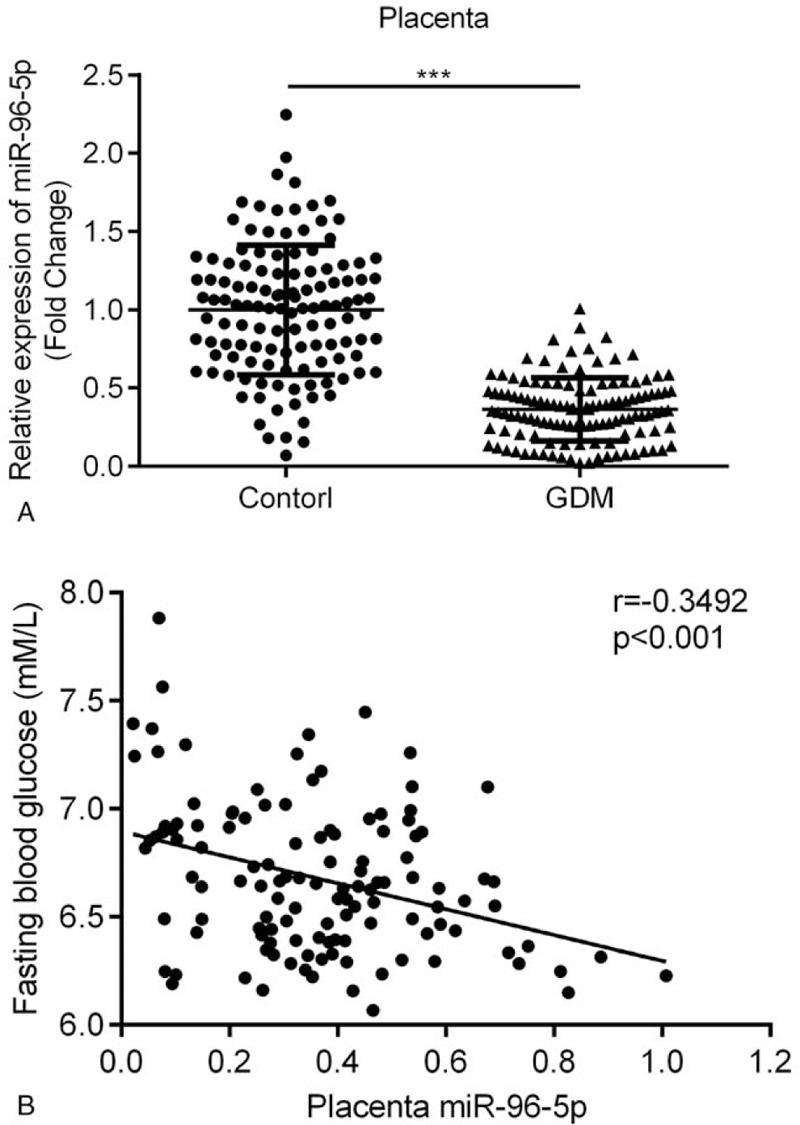
Comparison of miR-96-5p expression levels in placenta. (A) miR-96-5p expression in placenta samples from patients with gestational diabetes mellitus (GDM) and control participants. (B) Correlation between the miR-96-5p expression in placenta samples and fasting blood glucose levels of patients with GDM. ^∗∗∗^*P* < .001.

**Figure 2:**
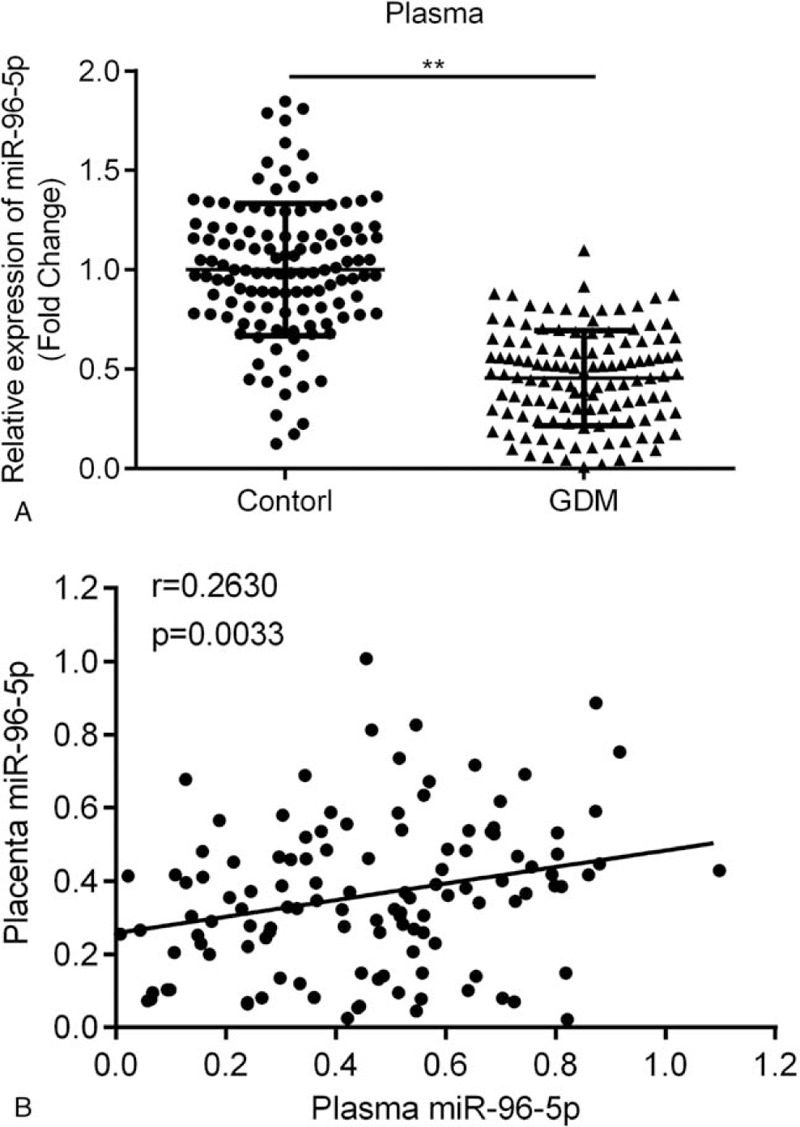
Comparison of miR-96-5p expression levels in plasma. (A) miR-96-5p expression levels in plasma samples from patients with gestational diabetes mellitus (GDM) and control participants. (B) Correlation between miR-96-5p expression levels in placenta and plasma samples from patients with GDM. ^∗∗^*P* < .01.

RT-qPCR was performed to compare the miR-96-5p expression levels in the placenta and plasma samples from control participants and patients with GDM. miR-96-5p expression levels were markedly low in the placenta samples (Fig. 1A, *P* < .001) from patients with GDM in comparison to those from control participants. Pearson correlation analysis indicated that miR-96-5p expression is negatively correlated with the fasting blood glucose levels of the patients (Fig. 1B, *r* = –0.3492, *P* < .001). Moreover, miR-96-5p expression levels in the plasma samples (Fig. 2A, *P* < .01) from patients with GDM were also low, and a strong positive correlation between miR-96-5p levels in the placenta and plasma of patients with GDM was identified (Fig. 2B, *r* = 0.2630, *P* = .0033). As shown in Table 1, significant differences in pre-pregnancy body mass index (BMI), fasting plasma glucose, 1-hour plasma glucose, and 2-hours fasting plasma glucose levels between control participants and patients with GDM were present (*P* < .0001). No significant differences in age, gestational age, and parity between the 2 groups were determined.

**Table 1:** Clinical characteristics of healthy pregnancies and GDM patients.

Factors (mean ± SD)	Healthy (n = 123)	GDM patients (n = 123)	*P*-value
Age, y	30.84 ± 2.95	31.23 ± 2.31	.2495
Gestational age, wks	39.52 ± 2.47	39.35 ± 3.81	.6783
Parity (times)	1.22 ± 0.52	1.26 ± 0.63	.5876
Pre-pregnancy BMI, kg/m^2^	21.25 ± 3.05	27.84 ± 3.99	<.0001
Fasting plasma glucose, mM	4.46 ± 0.38	5.57 ± 0.49	<.0001
1 hour plasma glucose, mM	6.42 ± 0.29	10.25 ± 0.53	<.0001
2 hours plasma glucose, mM	5.58 ± 0.36	8.48 ± 0.22	<.0001
Placenta miR-96-5p level (fold change)	1.00 ± 0.04	0.36 ± 0.02	<.0001
Plasma miR-96-5p level (fold change)	1.00 ± 0.03	0.46 ± 0.02	<.0001

### 3.2 miR-96-5p is a potential diagnostic biomarker for GDM

**Figure 3:**
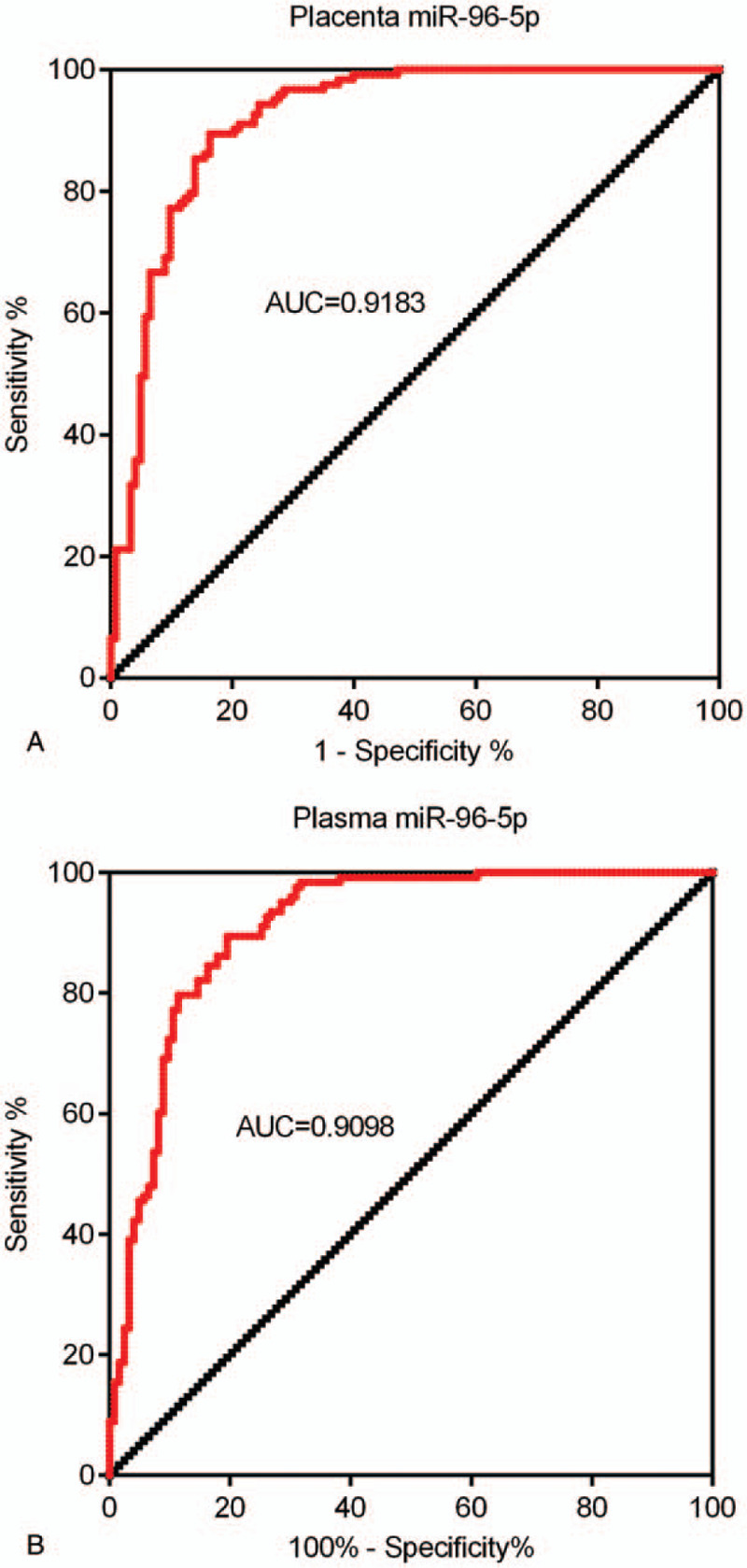
miR-96-5p is a potential diagnostic biomarker for gestational diabetes mellitus (GDM). (A) ROC curve of miR-96-5p expression levels in placenta samples from patients with GDM and control participants. (B) ROC curve of miR-96-5p expression levels in plasma samples from patients with GDM and control participants. ROC = receiver operating characteristic.

ROC curves were established to evaluate the potential of miR-96-5p expression in placenta and plasma miR-96-5p expression levels to distinguish GDM patients from healthy subjects. Results indicated that the area under the curve (AUC) for placenta and plasma miR-96-5p was 0.9183 (Fig, 3A, 95% confidence interval [CI] 0.8825–0.9541, cut-off value = 0.5937, sensitivity = 89.43%, and specificity = 83.74%) and 0.9098 (Fig. 3B, 95% CI 0.8723–0.9472, cut-off value = 0.7280, sensitivity = 86.18%, and specificity = 82.11%), respectively, suggesting that miR-96-5p levels in either placenta or plasma samples can serve as a diagnostic biomarker for GDM.

### 3.3 High glucose conditions result in decreased miR-96-5p expression levels in HRT-8/SVneo cells

**Figure 4:**
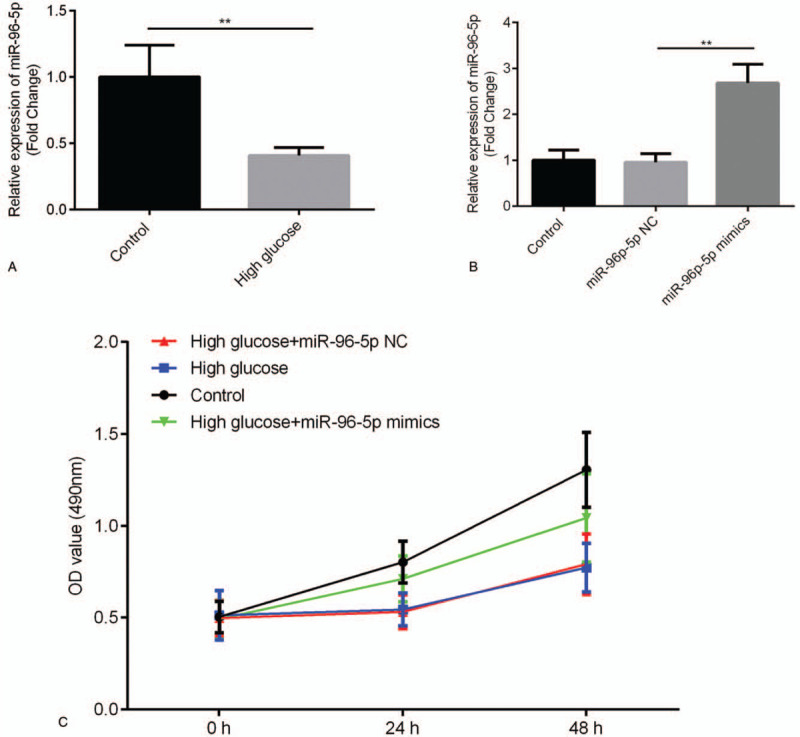
miR-96-5p can exert effects on the cell viability of trophoblast cells in vitro. (A) Effect of high glucose conditions on miR-96-5p expression in trophoblast cells in vitro. (B) Effect of miR-96-5p mimics on miR-96-5p expression in trophoblast cells in vitro. (C) Effect of miR-96-5p mimics on the viability of trophoblast cells under high glucose conditions in vitro. ^∗∗^*P* < .01.

HRT-8/SVneo cells were cultured under high glucose conditions, and miR-96-5p expression in HRT-8/SVneo cells under high and normal glucose conditions were compared. As shown Fig. 4A, miR-96-5p expression levels was markedly reduced in the cells cultured under high glucose conditions (*P* < .01).

### 3.4 miR-96-5p alleviates high glucose condition-induced reduction in the cell viability of HRT-8/SVneo cells

To investigate the function of miR-96-5p in the pathogenesis of GDM, miR-96-5p mimics were transfected into HRT-8/SVneo cells cultured under high glucose conditions, and MTT assay was performed. As shown in Fig. 4B, miR-96-5p mimics markedly increased miR-96-5p expression in HRT-8/SVneo cells (*P* < .01). The results of MTT assay indicated that high glucose conditions reduced the viability of HRT-8/SVneo cells (Fig. 4C) and that miR-96-5p mimics partially increased the viability (Fig. 4C) of HRT-8/SVneo cells cultured under high glucose conditions in vitro.

## 4 Discussion

In the present study, the potential roles and related mechanisms of miR-96-5p in GDM were explored. miR-96-5p expression levels were markedly low in both placenta and plasma samples from patients with GDM, and miR-96-5p can regulate the viability of trophoblast cells.

The aberrant expression of miRNAs in GDM has been reported in many previous studies; Wang et al^[24]^ reported that miR-657 can affect the polarization of macrophages into M1 type in GDM. Zhao and Tao^[18]^ suggested that miR-221 plays a protective role in GDM by regulating the expression of PAK1. Li et al^[22]^ reported that miR-96-5p expression was significantly decreased in placenta samples of from patients with GDM. In this study, miR-96-5p expression were markedly low in placenta samples from patients with GDM; this finding is consistent with that of Li et al.^[22]^ Moreover, miR-96-5p expression levels were negatively correlated with fasting blood glucose levels of patients with GDM, thereby indicating that miR-96-5p is related to disease severity. Altogether, our data indicate that miR-96-5p is a factor in the development of GDM.

The results of ROC curve analysis indicated that the miR-96-5p expression levels in placenta samples can be a factor for distinguishing between women with GDM and healthy individuals. Our data suggested the potential of miR-96-5p as a diagnostic marker for GDM; however, the challenges in obtaining placentas before childbirth may be a limiting factor for potential clinical application. Nevertheless, previous studies have suggested that miRNAs can be released by cells into the blood, and interestingly, unlike most protein biomarkers, circulating miRNAs can remain stable in the peripheral blood.^[25,26]^ Many previous studies have also proposed the roles of circulating miRNAs for the early diagnosis of various diseases,^[25–28]^ including GDM.^[19]^ In the present study, consistent with the expression pattern in placentas, miR-96-5p expression levels were markedly decreased in plasma samples from patients with GDM, and miR-96-5p expression levels in placenta and plasma samples from patients with GDM were positively correlated. Furthermore, the AUC of circulating miR-96-5p levels was 0.9183, which is similar to that of placenta miR-96-5p levels (0.9098). Therefore, the above data indicated that miR-96-5p levels in plasma may serve as a biomarker for the early diagnosis of GDM.

Trophoblast cells reportedly participate in the development of GDM.^[29,30]^ In the present study, high glucose conditions induced a marked decrease in miR-96-5p expression, suggesting that miR-96-5p may affect the activity of trophoblast cells in GDM. The decreased viability of trophoblast cells owing to high glucose conditions was considered an important factor for GDM.^[30–32]^ Moreover, our data indicated that high glucose conditions reduced the viability of trophoblast cells, which is consistent with previous findings. More importantly, miR-96-5p mimics can alleviate high glucose conditions-induced anti-proliferative effects in trophoblasts. Altogether, our findings suggest that miR-96-5p play a protective role in the development of GDM by regulating the viability of trophoblast cells.

In conclusion, miR-96-5p expression levels were markedly decreased in patients with GDM and is a potential diagnostic biomarker. Moreover, miR-96-5p can exert effects on the cell viability of trophoblast cells. Our data suggest that miR-96-5p has clinical significance in diagnosing and treating GDM.

## Author contributions

**Conceptualization:** Hongbo Qi.

**Data curation:** Xinyang Yu, Zhengfei Liu, Jie Fang.

**Formal analysis:** Xinyang Yu, Zhengfei Liu.

**Investigation:** Xinyang Yu.

**Methodology:** Xinyang Yu, Jie Fang.

**Software:** Zhengfei Liu, Jie Fang.

**Supervision:** Hongbo Qi.

**Validation:** Zhengfei Liu.

**Writing – original draft:** Hongbo Qi.

**Writing – review & editing:** Xinyang Yu, Jie Fang.
